# Heart Failure in Patients with Preserved Ejection Fraction: Questions Concerning Clinical Progression

**DOI:** 10.3390/jcdd3030027

**Published:** 2016-09-08

**Authors:** George E. Louridas, Katerina G. Lourida

**Affiliations:** Department of Cardiology, University General Hospital AHEPA, Aristotle University, Thessaloniki 54124, Greece; katerina.lourida@gmail.com

**Keywords:** heart failure, heart failure with preserved ejection fraction, left ventricular remodeling, heart failure progression, clinical phenotypes of heart failure

## Abstract

Over the last two decades, important advances have been made in explaining some pathophysiological aspects of heart failure with preserved ejection fraction (HFpEF) with repercussions for the successful clinical management of the syndrome. Despite these gains, our knowledge for the natural history of clinical progression from the pre-clinical diastolic dysfunction (PDD) until the final clinical stages is significantly limited. The subclinical progression of PDD to the clinical phenotype of HFpEF and the further clinical progression to some more complex clinical models with multi-organ involvement, similar to heart failure with reduced ejection fraction (HFrEF), continue to be poorly understood. Prospective studies are needed to elucidate the natural history of clinical progression in patients with HFpEF and to identify the exact left ventricular remodeling mechanism that underlies this progression.

## 1. Introduction

The clinical phenotype of patients with reduced ejection fraction (HFrEF) is extensively studied and understood, but the clinical phenotype and natural history of patients with preserved ejection fraction (HFpEF) remain poorly defined. There is incomplete knowledge and understanding of HFpEF pathophysiology. The relevant clinical trials are suboptimal in their design, and the treatment to a great extent is empiric [1]. The number of patients with HFpEF is increasing, partly related to the diastolic left ventricular (LV) dysfunction due to aging and to contributing comorbidities [2]. The comorbidities have a marked effect on the diastolic LV function and on the appearance of the classical clinical features of heart failure (HF) syndrome, in both phenotypes, HFrEF and HFpEF. The objective of this review is to state the undefined natural clinical progression of the HFpEF syndrome when the natural history of the HFrEF is well known to have a relentless clinical progression. Thus, this paper is “focused” mainly on the relatively unknown “clinical progression of HFpEF syndrome” and its relation to the diastolic dysfunction of the LV. Probably the identification of the left ventricular remodeling mechanism that underlies the HFpEF would help to understand the natural history of the syndrome.

## 2. Clinical Phenotypes of Heart Failure Based on the Ejection Fraction

During the last two decades emerged a new nomenclature of the clinical congestive HF syndrome that is based on the ejection fraction. This classification categorizes the patients with HF into those with HFrEF (EF < 50%) and those with HFpEF (EF > 50%). In the community, the prevalence of both phenotypes of HF, HFpEF and HFrEF, is equal [3]. HFrEF is characterized by loss of contractile myocardial force, while HFpEF is due to diastolic dysfunction [4]. The symptoms, clinical signs and physical findings are similar in both groups of patients. In a recent cohort study of 2166 adult outpatients with HF, with three-year follow-up, the clinical characteristics and outcomes of these patients were investigated [5]. Of these patients, 350 from the cohort of 2166 (16, 2%) with originally reduced (<40%) left ventricular ejection fraction (LVEF) demonstrated later on, during the follow-up, an improved or recovered ejection fraction (>40%). This group of patients with recovered ejection fraction had “a different clinical course than patients with HFpEF and HFrEF, with lower mortality, less frequent hospitalizations, and fewer composite end points” [5]. This paper emphasizes the overlapping of the HF clinical phenotypes.

To a number of clinicians, in view of the fact that the majority of the patients with HFrEF display to some extent diastolic dysfunction, the clinical distinction between the two syndromes is considered unnecessary [6]. According to this view, it is rather appropriate for the two entities to be considered as a continuous disease spectrum of overlapping clinical entities (phenotypes) [6]. To the majority of clinicians, because of different primary pathophysiological processes, the two HF syndromes should be considered as separate clinical phenotypes. This position is taken regardless of the clinical overlap between them. Moreover, separate clinical management is important due to distinct therapeutic and preventive factors. At the present state of knowledge, the symptomatic therapy of the two clinical entities is similar. Probably future clinical studies and objective diagnostic criteria will shed some light on a more specific treatment.

In exploring the fundamental molecular mechanisms of HFpEF, it is important to define the exact molecular processes responsible for the slowing of myocardial relaxation and for the increase of elastic stiffness in both the myocardium and the arterial wall of the peripheral arteries. In hypertension, the increased left ventricular afterload is a stimulus for ventricular structural remodeling and stiffening. These structural changes are leading to higher systolic left ventricular wall stress and slowing of ventricular relaxation [7]. The normal age-dependent gradual elevation in the arterial systolic pressure and pulse pressure are caused by vascular remodeling and stiffening of the arterial wall. These changes in the arterial wall are associated with a shift in left ventricular filling from early to late diastole [8]. The filling pattern of the elderly is similar to the filling pattern of the patients with heart disease who have diminished left ventricular relaxation and ventricular hypertrophy.

It is important to differentiate the name ‘diastolic dysfunction’ from the term of ‘diastolic heart failure’ syndrome. The age-dependent diastolic dysfunction does not necessarily indicate people with an increased risk of diastolic HF [8]. The pre-clinical diastolic dysfunction (PDD) is still a poorly understood subclinical condition. It demonstrates marked increase in all-cause mortality having the potentiality to progress from PDD to symptomatic HF [9,10].

The rising levels of biomarkers, brain natriuretic peptide (BNP) and the N-terminal pro-B-type natriuretic peptide (NT-proBNP), reinforce the clinical diagnosis of HFpEF. Their high negative predictive value excludes the diagnosis in some patients with suspected HF [11]. However, generally, the natriuretic peptides do not have sufficient diagnostic power to support the presence of diastolic dysfunction [11].

## 3. Clinical Progression in Patients with Preserved Ejection Fraction

**Figure 1:**
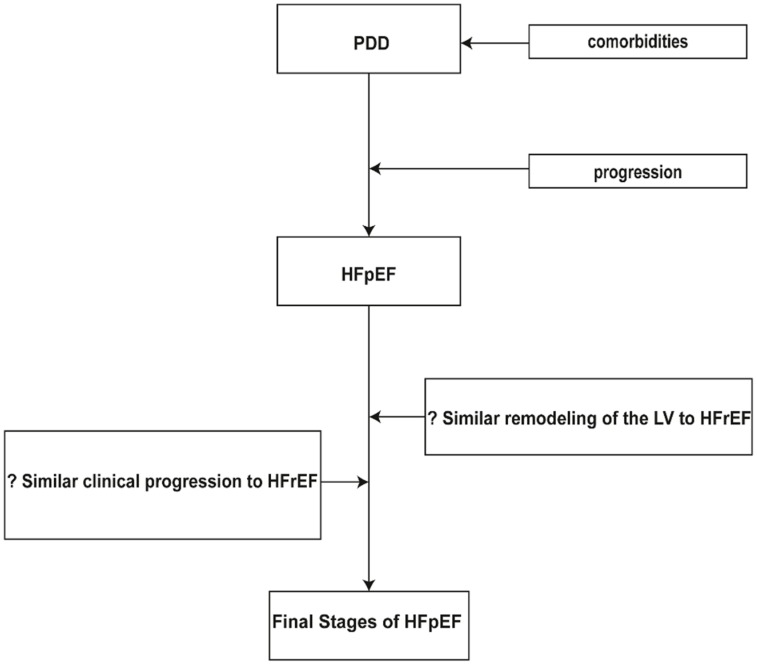
Progression of HFpEF. PDD = preclinical diastolic dysfunction; LV = left ventricle; HFpEF = heart failure with preserved ejection fraction; HFrEF = heart failure with reduced ejection fraction.

In general, very few original papers are addressed to the natural history of PDD and to the clinical progression from PDD to overt symptomatic HF [12]. Furthermore, there is limited literature for the clinical progression of established HFpEF from the initial symptomatic HF period to the final stages of HF (Figure 1). The majority of the patients with HFpEF do not show any particular hidden cardiac disease. Nevertheless, most of them present with one or more comorbidities: old age, pulmonary disease, diabetes mellitus, anemia, renal disease, obesity, sleep disordered breathing, coronary artery disease, hypertension, peripheral vascular disease and a variety of other non-cardiac comorbidities.

Some publications provided useful epidemiological facts to the preclinical prevalence of diastolic dysfunction in the general population [9,13]. The prevalence of asymptomatic diastolic dysfunction fluctuates between 20%–35% in the elderly population and increases with age and various comorbidities [14,15]. Most frequently, after the occurrence of PDD, the progression to symptomatic HFpEF is influenced by cardiovascular and non-cardiac risk factors. Patients with diabetes mellitus, coronary artery disease or hypertension complicated with PDD demonstrate a significantly higher risk to progress in HF compared with patients having the same comorbidities, but without the presence of PDD [10]. An established epidemiological fact is that in hypertensive patients, the efficient therapy of hypertension syndrome can prevent the progression of diastolic dysfunction to HFpEF [16].

In OCHFS (Olmsted County Heart Function Study), for a period of four years, changes were recorded in diastolic function in randomly-selected individuals, 45 years or older [17]. The trial was scheduled to affirm the relationship between diastolic dysfunction and the risk of the appearance of HF syndrome. Diastolic dysfunction prevalence increased over the initial four years, and in some individuals, during an additional follow-up period of six years, their established diastolic dysfunction progressed to HF [17].

In a cohort of PDD subjects, progression to clinical HF according to Framingham criteria was expected to be low with a two-year cumulative probability of 1.9% [18]. Moreover, there was a “moderate degree of progression to the development of symptoms and cardiac hospitalizations over 2 years (31.1%)” [18]. The same authors found that in patients with PDD, only peripheral vascular disease and hypertension were independently related to the development of symptoms.

The development of left ventricular diastolic dysfunction is linked to left ventricular hypertrophy (LVH). Yet, different studies in animals and humans indicate that the diastolic dysfunction starts before the appearance of LVH. Dupont et al. [19] demonstrated that in three-week-old spontaneously hypertensive rats, the onset of left ventricular diastolic dysfunction appears before LVH and therefore is not related to LVH or hypertension. At present, it is not clear if the regression of LVH or the improvement of the diastolic function would avert the progression from diastolic dysfunction to HFpEF [10]. We do not have robust data to support the hypothesis that the most probable mechanism of clinical progression in patients with HFpEF is the left ventricular remodeling mechanism, similar to that of HFrEF patients.

In a population-based study, 388 patients, mean age 67 ± years, with a prevalence of renal insufficiency (glomerular filtration rate <60 mL/min) of 34% and with a Grade 2–4 diastolic dysfunction in echocardiography, were followed-up over three years [20]. The objective was to determine the significance of the risk factors that are associated with the progression of PDD to symptomatic HF. The three-year cumulative probabilities of the development of HF, atrial fibrillation, cardiac hospitalization and mortality were 11.6%, 14.5%, 17.7% and 10.1% respectively. It was determined that age, right ventricular systolic pressure and renal dysfunction were independently associated with progression to HF.

Diabetic cardiomyopathy is linked to the presence of myocardial hypertrophy and stiffness that induce diastolic dysfunction. In diabetic patients, poor glycemic control is related to an increased risk of HF, but it remains unclear whether treating hyperglycemia will reduce progression or reverse the presence of HFpEF syndrome [21]. From et al. [22], in 1760 diabetic patients, with a tissue Doppler imaging of diastolic function, identified 411 patients (23%) with diastolic dysfunction. The above authors evaluated the outcomes of PDD in diabetic patients and demonstrated that in five years, the cumulative probability of HF for diabetic patients with PDD was 36.9% compared to 16.8% for patients without PDD [22].

Ren et al. [23] evaluated the association of PDD with cardiovascular effects in 693 patients with coronary heart disease and no history of HF. They discovered that 455 (66%) patients had normal left ventricular diastolic dysfunction, 166 (24%) had mild diastolic dysfunction and 72 (10%) had moderate to severe diastolic dysfunction. They concluded after multivariable adjustment that in patients with coronary heart disease, the presence of moderate to severe diastolic dysfunction was strongly predictive of incident hospitalization for HF and death from heart disease.

In the I-PRESERVE (Irbesartan in Heart Failure with Preserved Ejection Fraction Study) study, the mode of death in patients with HFpEF and the role of irbesartan to alter this possibility were examined [24]. It was proven that the mode of death in HFpEF patients was cardiovascular in 60% (including 26% sudden, 14% HF, 5% myocardial infarction and 9% stroke), non-cardiovascular in 30% and unknown in 10%. Treatment with irbesartan did not affect the overall mortality nor the distribution of mode-related mortality rates.

The present therapy of HFpEF has not been effective in reducing morbidity and mortality. Angiotensin-converting enzyme inhibitors (ACEI), angiotensin-II receptor antagonists (ARA), diuretics and beta blockers were proven ineffective to decrease the composite product of hospital readmission and death in patients with HFpEF [25,26,27,28]. In a review study of patients with HFrEF and HFpEF who participated in the DIG (Digitalis Investigation Group) [29] and the CHARM (Candesartan in Heart Failure: Assessment of Reduction in Mortality and Morbidity) [30] trials, the outcomes were compared between the two types of HF [31]. Patients in these trials, along with the I-PRESERVE study [26], were compared for age, sex distribution and comorbidity and provided interesting insights into the relation between phenotype, rates of death and HF hospitalization. The poor clinical outcomes in HFpEF patients were not explained by the presence of old age, gender, blood pressure or left ventricular structural remodeling; they were better explained by the presence of the clinical syndrome of HF. Ahmed et al. [32] found no effect of digoxin therapy on mortality in either HFpEF or HFrEF, but it was advantageous in the composite product of repeated hospitalizations or death. Furthermore, carvedilol therapy failed to improve patients with HFpEF [33].

The HOPE (Heart Outcomes Prevention Evaluation) study revealed that in patients having high cardiovascular risk, particularly patients with borderline hypertension, the risk of developing HF was reduced after therapy with ACEIs [34]. In the ALLHAT (Antihypertensive and Lipid-Lowering Treatment to Prevent Heart Attack Trial) trial, it was proven that diuretic therapy was more successful than calcium channel blockers for reducing HF [35]. There are some suggestions that the lowering of the high blood pressure in hypertensive patients may lead to the regression of the LV diastolic dysfunction [36,37]. In the Hong Kong diastolic HF study, in a population with HFpEF, treatment with diuretics, irbesartan and ramipril improved LV global and regional diastolic function and decreased levels of NT-proBNP [38]. The TOPCAT (Trial of Aldosterone Antagonist Therapy in Adults with Preserved Ejection Fraction Congestive Heart Failure) trial was designed to evaluate the role of spironolactone treatment on the morbidity, mortality and quality of life in patients with HFpEF [39]. It was concluded that the treatment with spironolactone did not alter the occurrence of the primary composite result of cardiovascular death, aborted cardiac arrest or hospitalization [40]. In another paper of the TOPCAT trial, it was demonstrated that in HFpEF patients from Russia/Georgia, there was no detectable impact of spironolactone on any clinical outcomes [41]. That is in contrast with the Americas where “the rates of the primary outcome, cardiovascular death, and hospitalization for heart failure were significantly reduced by spironolactone”. In another recent randomized trial, in patients with HFpEF, the treatment with spironolactone improved their LV diastolic function, but that did not have an impact on maximal exercise capacity, patient symptoms or quality of life [42].

A new HFpEF paradigm was presented recently with the emphasis moved from the left ventricular afterload increase to coronary microvascular inflammation. Paulus and Tschope [43], suggest that a systemic proinflammatory state induced by comorbidities leads to coronary microvascular endothelial inflammation, to an increase of reactive oxygen species (ROS) and to reduction of nitric oxide bioavailability for adjacent cardiomyocytes. Both of them limit protein kinase G (PKG) activity and cyclic guanosine monophosphate (cGMP) in cardiomyocytes. The PKG activity is lower in HFpEF patients than in patients with HFrEF and that is related to the greater size of the cardiomyocytes that are stiff and hypertrophied [44]. The reduced PKG activity increases resting tension of cardiomyocytes that favors hypertrophy and induces concentric left ventricular remodeling. In contrast, the normal functional activity of PKG puts an end to myocardial hypertrophy progression [45]. Both the stiff cardiomyocytes and the interstitial fibrosis cause diastolic left ventricular dysfunction and, finally, left ventricular stiffness and HF [43,46]. The cGMP-PKG pathway probably is a promising therapeutic target for patients with HFpEF, a probability “that is mainly based on its role in the phosphorylation of the giant cytoskeletal protein titin” [47,48]. The above relocation of interest to endothelial inflammation shifts attention to concentric LVH remodeling and diastolic dysfunction in contrast to the HFrEF remodeling, which is driven by progressive loss of cardiomyocytes [49]. Pellicori et al. [50] believe that left atrial emptying function (LAEF) is a better prognostic marker in outpatients referred for HF, while the improvement of the LA structural remodeling after therapy is associated with better clinical outlook in patients with HF [51].

## 4. Incomplete Knowledge of Clinical Progression

**Figure 2:**
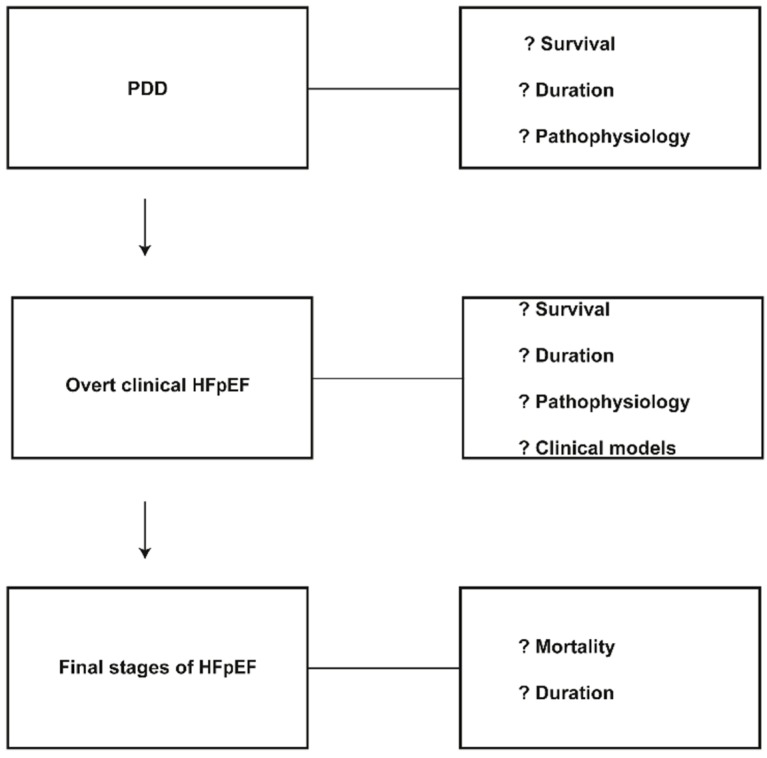
Phases of clinical progression of HFpEF.

The previously-recorded studies were limited in focus and clinical follow-up of patients with PDD and HFpEF. The subclinical progression of the PDD to the clinical entity of HFpEF and the further clinical advance to more complex clinical models of HF, analogous to HFrEF, continue to be poorly understood. Few large animal models that imitate the human HFpEF syndrome are described. Conceicao et al. in a recent article are suggesting that “although most of the heart failure animal models currently available represent heart failure with reduced ejection fraction, several HFpEF animal models have been proposed. However, few of these fulfill all the features present in human disease” [52]. The relevant clinical trials are suboptimal in their design, and the treatment to a great extent remains largely empiric [1]. The present limited natural history data on follow-up in all clinical stages of PDD and HFpEF patients raise a number of questions: (1) there is an unspecified relationship between PDD and progression to HFpEF; (2) there are no data for the relationship between the degree of PDD and clinical progression, as people with the same degree of PDD develop different clinical phenotypes; (3) there is an undetermined connection of the progression rate from PDD to HFpEF for each particular comorbidity; (4) there are no data for similarities or differences between the two clinical phenotypes on clinical deterioration and progression up to the final stages of HF; (5) there are no clear data on the mortality rate of HFpEF patients during the whole period of clinical deterioration up to the final stages; more data are required for the duration and survival in each step of the syndrome (Figure 2); (6) robust data are needed to support the notion that in HFpEF, the left ventricular remodeling is the most probable cause of the clinical decline; (7) it is speculated that the two phenotypes differ in their remodeling mechanism, and as a consequence, they have developed different clinical progression rates; (8) novel prognostic imaging biomarkers are required; probably, LA function is emerging as an important prognostic biomarker [51].

HFrEF syndrome has the characteristics of an entity with a progressive clinical deterioration with a relentless course toward end-stage myocardial failure with periods of clinical stabilization and periods of clinical instability [53]. At present, we are short of a similar clinical picture with the characteristics of a progressive and relentless course for HFpEF syndrome. The syndrome of HFpEF has a complex pathophysiology, heterogeneous etiology and is complicated by various comorbidities, which are probably predictors of clinical progression [54]. Ferrari et al. suggest that “the absence of clear diagnostic clinical criteria was the major barrier to progress” in understanding the syndrome of HFpEF, and there are “difficulties in performing randomized clinical trials, due to difficulties in characterizing HFpEF itself” [55]. Lekavich et al., believe that “longitudinal studies are lacking” and “studies designed and adequately powered to study the impact of race and age…to further incident HFpEF research” are needed [56].

Thus, in answer to the above questions, the availability of a more suitable model paradigm of clinical HFpEF progression is required. Furthermore, well-designed future longitudinal clinical trials are needed in order to validate specific subgroups of HFpEF phenotypes from the PDD phase up to the terminal stages. This approach will address better the complexity of HFpEF syndrome and will speed the development of novel diagnostic and therapeutic procedures.

## 5. Conclusions

There is limited information of the natural history and clinical progression of PDD and HFpEF syndromes. The HFpEF syndrome is a term that involves many distinct mechanisms of disease. Thus, diastolic function abnormalities may be driven more by calcium handling in one patient and tissue fibrosis in another and ventricular-aortic coupling in another. This has further complicated our ability to define the natural history and clinical progression of this heart failure syndrome. The insufficient clinical and epidemiological data prohibit the comparison of their natural history with this of HFrEF syndrome. It is also speculated that the HFpEF patients have a different LV remodeling mechanism than that of HFrEF patients. As a consequence of that, they have developed different clinical progression rates. Prospective studies are needed to describe the progressive nature of PDD and HFpEF syndromes. We need to be better at disease phenotyping in HFpEF in order to make progress in this area. LVEF alone will not be adequate.
